# Primer on machine learning applications in brain immunology

**DOI:** 10.3389/fbinf.2025.1554010

**Published:** 2025-04-17

**Authors:** Niklas Binder, Ashkan Khavaran, Roman Sankowski

**Affiliations:** Institute of Neuropathology, Faculty of Medicine, University of Freiburg, Freiburg, Germany

**Keywords:** single-cell genomics, data integration, deep learning, multi-omcis, development, trajectory analysis, gene co-expression network, perturbation prediction

## Abstract

Single-cell and spatial technologies have transformed our understanding of brain immunology, providing unprecedented insights into immune cell heterogeneity and spatial organisation within the central nervous system. These methods have uncovered complex cellular interactions, rare cell populations, and the dynamic immune landscape in neurological disorders. This review highlights recent advances in single-cell “omics” data analysis and discusses their applicability for brain immunology. Traditional statistical techniques, adapted for single-cell omics, have been crucial in categorizing cell types and identifying gene signatures, overcoming challenges posed by increasingly complex datasets. We explore how machine learning, particularly deep learning methods like autoencoders and graph neural networks, is addressing these challenges by enhancing dimensionality reduction, data integration, and feature extraction. Newly developed foundation models present exciting opportunities for uncovering gene expression programs and predicting genetic perturbations. Focusing on brain development, we demonstrate how single-cell analyses have resolved immune cell heterogeneity, identified temporal maturation trajectories, and uncovered potential therapeutic links to various pathologies, including brain malignancies and neurodegeneration. The integration of single-cell and spatial omics has elucidated the intricate cellular interplay within the developing brain. This mini-review is intended for wet lab biologists at all career stages, offering a concise overview of the evolving landscape of single-cell omics in the age of widely available artificial intelligence.

## 1 Introduction

The field of brain immunology has undergone a remarkable transformation in recent years, challenging the traditional view of the brain as an “immune-privileged” site (Louveau et al., 2015). Researchers are now beginning to appreciate the complex and dynamic immune cell landscape in the brain that plays crucial roles in both health and disease (Prinz and Priller, 2017; Castellani et al., 2023). This evolving understanding has been largely driven by technological advancements, particularly in single-cell and spatial technologies, which have enabled detailed characterization of immune cell heterogeneity and spatial organization within the central nervous system (CNS) (Masuda et al., 2020; Mrdjen et al., 2018).

A large portion of research in the field of brain immunology has focused on myeloid cells, especially microglia and CNS- or border-associated macrophages (CAMs or BAMs), due to their critical role in brain homeostasis and pathology (Prinz et al., 2021; Van Hove et al., 2019). Single cell omics has facilitated the discovery of cell types and cell states, mapping of the dynamic immune landscape, and elucidation of complex cellular interactions in various neurological disorders (Sankowski et al., 2019; Jordão et al., 2019; Sankowski et al., 2022). However, as the complexity of the data generated by these methods increases, significant computational and analytical challenges arise. State-of-the-art omics experiments profile tens of thousands up to millions of cells across multiple modalities–in the tissue context or cell suspensions–generating datasets of extraordinary scale and complexity. Traditional statistical methods only superficially capture the intricate structures inherent in these high-dimensional datasets (Lähnemann et al., 2020; Svensson et al., 2020). In addition, the immense volume of data often pushes the boundaries of current hardware, demonstrating the need for novel approaches to data processing and analysis (Melsted et al., 2021).

In response to these challenges, machine learning, (see glossary in Table 2) especially deep learning (see glossary in Table 2) approaches, have emerged as powerful tools for the analysis of complex single-cell datasets (Eraslan et al., 2019; Li et al., 2020). For example, machine learning tools can be used to enhance dimensionality reduction and integration of large datasets, but also for more sophisticated tasks, such as predictive modeling of gene perturbations (Xu et al., 2021; Lotfollahi et al., 2022; Roohani et al., 2024). Machine learning-based methods have shown great potential in capturing patterns and non-linear relationships within high-dimensional biological data (Ching et al., 2018).

This review aims to provide a concise overview of recent advances in single-cell omic data analysis and discuss their applicability in brain immunology, with a particular focus on machine learning techniques. We will describe the evolution of data analysis methods, from conventional approaches to novel deep learning frameworks, such as variational autoencoders (see glossary in Table 2), graph neural networks, and emerging foundation models (see glossary in Table 2). With a focus on brain development, we highlight how single-cell analyses have unveiled immune cell heterogeneity, identified temporal differentiation trajectories and uncovered potential therapeutic targets for various pathologies.

## 2 Conventional analysis methods in single-cell omics

The advent of single cell technologies has transformed our understanding of the composition and states of brain immune cells. Two prominent computational frameworks emerged as cornerstones of single-cell analysis: Seurat (Stuart and Satija, 2019) and Scanpy (Wolf et al., 2018). Seurat and Scanpy, developed for R and Python, respectively, incorporate essential statistical techniques adapted for single-cell data. The analysis workflows are consistent between both programs with some notable differences (Rich et al., 2024). To account for technical variations in sequencing depth between cells and to stabilize variance, the analysis typically begins with normalization and log transformation (Hafemeister and Satija, 2019; LunA. T. et al., 2016). In the feature selection step, highly variable genes are selected for downstream analysis. Then, dimensional reduction is applied to simplify the data structure using deterministic algorithms, like principal component analysis. Next, cell similaritiesare quantified.

Originally, when datasets were relatively small (typically <5,000 cells), cell similarities were calculated using Euclidean distances. Since the resulting matrices did not scale well for larger datasets above 10,000 cells, more recent workflows are based on nearest-neighbor graphs to quantify cell similarity (see glossary in Table 2). These graphs are underlying downstream analyses, including cell clustering (see glossary in Table 2) and embedding into euclidean space with Uniform Manifold Approximation and Projection (UMAP) (see glossary in Table 2). While Seurat and Scanpy both rely on this graph-based strategy, their graphs are constructed differently, leading to marginal different UMAP representations and clustering results between these two frameworks (Rich et al., 2024). Despite enabling major biological breakthroughs, several shortcomings emerged, including dependency on dimensionality reduction and highly variable gene selection. Crucial biological information may be lost in the process. To address this bias, advanced machine learning algorithms have been adopted for single-cell omics.

## 3 Advanced machine learning approaches

Deep learning, a subset of machine learning, employs deep neural networks with multiple layers to learn and represent complex data patterns. Inspired by the structure and function of the human brain, these networks consist of interconnected nodes (neurons) that process and transmit information (LeCun et al., 2015). The impact of deep learning models on various domains of biological research has been profound, particularly in single-cell omics, image analysis, and protein structure prediction (Ching et al., 2018). A prime example is AlphaFold, developed by DeepMind, which has revolutionized protein structure prediction (Jumper et al., 2021). AlphaFold uses attention-based neural networks to predict three-dimensional protein structures from amino acid sequences with high accuracy. This breakthrough has had significant implications for understanding protein function, drug discovery, and disease mechanisms, which ultimately led to the award of the Nobel Prize in Chemistry 2024 (Callaway, 2024).

**FIGURE 1:**
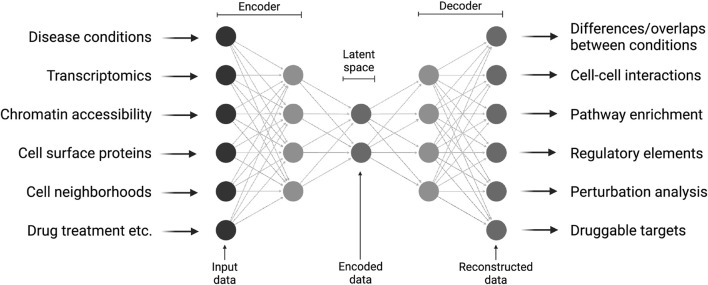
Multi-modal integration of single-cell data using deep learning. Various molecular and clinical information is provided to a deep neural network that is capable of learning a lower-dimensional representation of the data and performing complex predictions on new data. Created in BioRender. Sankowski, R. (2025) https://BioRender.com/e37u167.

Deep learning models have enhanced single-cell omics by enabling the identification of complex features directly from raw, high-dimensional datasets, minimizing the need for extensive pre-processing (Eraslan et al., 2019). This capability has facilitated the development of powerful tools for critical tasks such as dimensionality reduction, batch correction, and data integration. As the field has evolved, various models have emerged, each addressing specific challenges in single-cell data analysis (Erfanian et al., 2023). Among these, one of the most influential is scVI (Single-cell Variational Inference) (Lopez et al., 2018). scVI, a variational autoencoder, learns a probabilistic representation of gene expression data while accounting for technical factors such as batch effects and library size. Autoencoders have also been adapted to integrate and jointly represent multiple modalities such as RNA, surface protein expression, chromatin accessibility, and spatial context (Gayoso et al., 2021; Lopez et al., 2022; Ashuach et al., 2023). This is a particularly useful aspect of single-cell omics. By projecting complementary cell information into a so-called latent space via an encoder-decoder architecture, this approach can be used to obtain unseen information from new datasets, like the reaction to drug treatments or the prediction of gene perturbations (Figure 1).

These developments in single-cell omics and protein structure prediction exemplify how deep learning is transforming our ability to extract meaningful insights from complex biological data. As the field progresses, researchers are now exploring more generalized approaches, aiming to create models that can be applied across a wide range of biological questions and datasets. This shift has led to the emergence of foundation models in single-cell omics, which promise to revolutionize our understanding of cellular biology and gene regulation.

## 4 Foundation models in single-cell and spatial omics

A foundation model is a type of artificial intelligence system (see glossary in Table 2) that is trained on a large collection of data and can be fine-tuned for a variety of downstream tasks, such as language processing, computer vision, and speech recognition (Bommasani et al., 2022; Naveed et al., 2024). These models leverage self-supervised learning on vast datasets to develop contextual representations that can be adapted to specific applications. Foundation models have recently gained traction in the field of single-cell omics due to their ability to leverage large-scale datasets and transfer learning capabilities (Schaar et al., 2024; Boiarsky et al., 2023). These models have shown promise in various applications within single-cell biology, including cell type classification, gene expression prediction, and cross-modality integration (Cui et al., 2024).

Dozens of foundation models have emerged in the single-cell omics field over the past 3 years. We highlight six representative dissociated single-cell models selected based on citation impact, methodological innovation, and demonstrated applications: scBert (Yang et al., 2022), Geneformer (Theodoris et al., 2023), scGPT (Cui et al., 2024), Universal Cell Embeddings (Rosen et al., 2024), scFoundation (Hao et al., 2024) and CellFM (Zeng et al., 2024), along with two spatial models, Nicheformer (Schaar et al., 2024) and scGPT-spatial (Wang et al., 2025), which incorporate spatial information. Table 1 provides a broader overview of current foundation models, including these highlighted examples and additional notable contributions to the field.

**TABLE 1:** Overview of selected current foundational models in Single-Cell and Spatial Omics. Models were selected based on citation impact, methodological innovation, and demonstrated applications. The table includes both models described in detail in the text and additional contributions to provide broader context of this field.

Model	Claims	References
scBERT	Leverages large-scale unlabeled scRNA-seq data to capture gene-gene interactions and supports fine-tuning on specific datasets for cell type annotation	Yang et al. (2022)
Geneformer	Makes various predictions in the context of network biology, even when limited data is available	Theodoris et al. (2023)
scGPT	Enables the integration of multiple modalities and predicts perturbation responses and infers gene-interactions and gene regulatory networks	Cui et al. (2024)
scFoundation	Can be fine-tuned to infer gene modules and predict the response of single-cells and tissues to genetic and drug perturbations	Hao et al. (2024)
Universal Cell Embeddings	Trained on multiple species, maps species not in training data without fine-tuning	Rosen et al., 2023 (2024)
CellFM	Largest model. Predicts gene function, cell type annotation, perturbation effects, and gene networks	Zeng et al. (2024)
GeneCompass	Predicts cell fate transition and gene homologies across human and murine cells	Yang et al. (2024)
tGPT	Applied to bulk tissue sequencing samples to extract features associated with genomic alterations and immunotherapy response	Shen et al. (2023)
CELLama	Supports flexible applications ranging from cell typing to the analysis of spatial contexts	Choi et al. (2024)
Nicheformer	Enables zero-shot analysis of single-cell and spatial data in human and murine cells	Schaar et al. (2024)
scGPT-spatial	Pretrained on 30 M spatial transcriptomic profiles with protocol-aware decoding and neighborhood-based training to capture spatial context	Wang et al. (2025)

scBERT (single-cell Bidirectional encoder representations from transformers) is a pretrained deep neural network-based model. It addresses the limitations of existing cell type annotation methods by leveraging large-scale unlabeled scRNA-seq (see glossary in Table 2) data to capture gene-gene interactions and subsequently fine-tuning on specific datasets for cell type annotation. The authors report that this approach enables scBERT to demonstrate superior performance in cell type annotation, novel cell type discovery, and robustness to batch effects, while also offering improved model interpretability compared to traditional methods (Yang et al., 2022).

**TABLE 2:** Glossary of technical terms.

Term	Definition
Model	A computational system designed to find patterns in data and make predictions or decisions based on those patterns
AI	Artificial Intelligence, a field of computer science focused on creating systems that can perform tasks typically requiring human intelligence, such as pattern recognition, decision making, and prediction. Now widely adopted in medicine and biology
Machine Learning	A subset of AI that enables systems to automatically learn and improve from experience without being explicitly programmed, by identifying patterns in data to make predictions or decisions (Greener et al., 2022)
Deep Learning	A specialized form of machine learning using neural networks with multiple layers (deep architectures) to automatically learn hierarchical representations of data, particularly effective for complex tasks like image recognition and gene expression analysis (LeCun et al., 2015; Eraslan et al., 2019)
Foundation Model	A large artificial intelligence system trained on vast amounts of data that can be adapted for various specific tasks through fine-tuning Bommasani et al. (2022)
UMAP	Uniform Manifold Approximation and Projection, a technique for representing high-dimensional data in a lower-dimensional space while preserving important relationships between data points (McInnes et al., 2018)
Clustering	A computational process that groups cells with similar properties (such as gene expression patterns) into distinct clusters during single-cell analysis, allowing researchers to identify and characterize different cell populations (Kiselev et al., 2019)
Graph (SNN/KNN Graph)	A network structure where nodes (cells) are connected to their most similar neighbors based on gene expression patterns, used to identify cell relationships
scRNA-seq	Single-cell RNA sequencing, a technology that measures the amount of gene activity in individual cells, providing detailed insights into cellular heterogeneity
Autoencoder	A neural network architecture that learns to compress data into a compact representation and then reconstruct it, useful for finding essential patterns in complex biological data
Transformer	A neural network architecture that processes sequential data using self-attention mechanisms, allowing it to capture relationships between different elements in the sequence regardless of their distance from each other. Originally developed for natural language processing but now widely used across many domains

Geneformer is a context-aware, attention-based deep learning model pre-trained on 30 million single-cell transcriptomes. By transfer learning it can make various predictions in the context of network biology, even when limited data are available. For instance, the authors report that the model can be fine-tuned to predict gene dosage sensitivity and chromatin dynamics. Moreover, the model enables prediction of changes in network dynamics in response to gene deletion or treatments *in silico* (Theodoris et al., 2023).

scGPT (single-cell Generative Pre-trained Transformer) (see glossary in Table 2) is another foundation model, which has been pre-trained on 33 million human cells from various tissues. The model enables the integration of multiple modalities and predict perturbation responses (Cui et al., 2024). In addition, the authors claim that the model can be used to infer gene-interactions and gene regulatory networks. A model like scGPT can be utilized in two distinct settings: fine-tuned and zero-shot. In the fine-tuned setting, the pre-trained model is further trained on task-specific data, while in the zero-shot setting, the model is applied directly to new tasks without any additional training to make predictions (Cui et al., 2024).

Universal Cell Embeddings is a model that can analyze gene expression data across multiple different biological species (such as human, mouse, and other organisms). The authors claim that it can process and represent new single-cell RNA sequencing datasets without requiring additional training or fine-tuning. It converts RNA sequencing data into protein embeddings, which allows the model to effectively cluster and classify cells from species that were not included in its original training data (Rosen et al., 2023; 2024).

scFoundation has been trained on 50 million cells from various tissues and can be fined-tuned to infer gene modules and predict the response of single-cell and tissues to genetic and drug perturbations. Furthermore, the authors report that their model can improve clustering results by enhancing the read depth of cells in a setting without any fine-tuning (Hao et al., 2024).

At the moment, the largest model is CellFM trained on 800 million parameters from 100 million cells from various tissues. The authors of cellFm claim that the model can predict gene function prediction, cell type annotation, perturbation effect prediction, and gene network analysis. (Zeng et al., 2024).

While these foundation models have advanced single-cell analysis capabilities, they primarily focus on transcriptomic data without incorporating spatial context. More recently, spatially aware foundation models have emerged. These models leverage information from both dissociated and spatial transcriptomics data. Nicheformer (Schaar et al., 2024) is pretrained on an extensive dataset of over 57 million dissociated cells and 53 million spatially resolved cells across 73 tissues from both human and mouse. The model enables novel applications such as predicting the spatial context of dissociated cells, effectively transferring spatial information to traditional scRNA-seq datasets (Schaar et al., 2024).

scGPT-spatial (Wang et al., 2025) extends the scGPT model by including spatial information through continual pretraining on SpatialHuman30M, a dataset containing 30 million spatial transcriptomes. Its key innovations include a Mixture of Experts decoder that automatically handles different data formats and training methods that recognize how cells physically relate to each other in tissues. These advances allow the model to effectively combine different types of spatial data, identify cell types within mixed samples, and accurately predict gene expression based on a cell’s location context—all with better results than previous methods (Wang et al., 2025).

## 5 Case study brain development

Single-cell studies provide high-resolution information on cell types and cell states present in a complex system at the time of measurement. Brain development is a particularly dynamic period in mammals due to adaptive and rapid processes that include formation of synapses, cell differentiation and establishment of neural circuits (Stiles and Jernigan, 2010). Although it may be challenging to assess the molecular processes that occur at any given time in a developing human, single-cell studies of macrophages provide a particularly valuable use case. Macrophages are found in virtually all organ systems at any given time in life. As highly dynamic cells, macrophages are imprinted by the respective tissue they reside in (Guilliams and Svedberg, 2021). Thus, by mirroring their surroundings, macrophages are quite informative about the physiology of a developing human brain.

**FIGURE 2:**
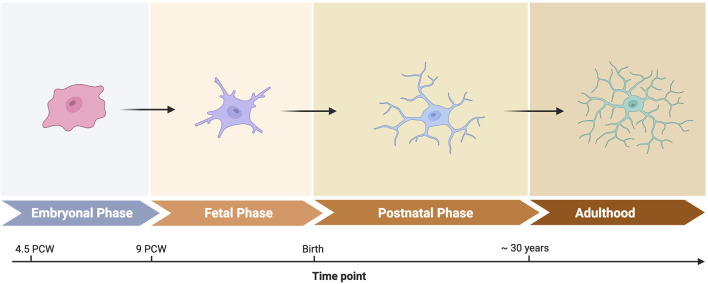
Microglia phenotypes throughtout development. Created in BioRender. Sankowski, R. (2025) https://BioRender.com/r23c965.

Several single-cell studies examine the developing human brain, witha focus on broader aspects of brain development (Eze et al., 2021; Zeng et al., 2023; Braun et al., 2023), and two studies specifically focused on brain macrophages (Kracht et al., 2020; Sankowski et al., 2024). The studies analyze the late embryonal and early fetal periods between the 5th and 23rd weeks post conception. They show that human microglia undergo major maturation steps as the brain tissue around them matures. During this period, microglia phenotypes evolve to resemble mature microglia (Figure 2). However, microglia and CAMs retain a clear distinction from adult brain macrophages. This distinction is exemplified by an increased expression of the iron scavenging surface marker CD71 that is encoded by the *TFRC* gene (Sankowski et al., 2024). One possible interpretation is reduced oxygen availability in the fetus, leading to a critical need for iron for oxygen transport. These findings identify critical phases during human brain development and explain immune cell phenotypes in the context of dramatic changes in surrounding brain tissue.

## 6 Challenges and outlook

The rapid development of models in the analysis of multi-omics data in recent years seems promising. While in using conventional analysis methods the field coalesced around Seurat and Scanpy, best practices for using the foundational models have as of yet not been widely adopted. Benchmarks in the field have not yet been established for a better comparison between models (Ding et al., 2024; Fu et al., 2024). Current models are often “black boxes”, and improvements in interpretability will help to deepen understanding of the underlying biology (Talukder et al., 2021). One potential obstacle is finding the right hyperparameters for each model, as identifying optimal configurations requires extensive experimentation. Even small changes in parameters can lead to significant differences in biological interpretability (Theodoris, 2024).

Current multi-omics approaches primarily focus on RNA-seq data, with only a few incorporating proteomics and spatial sequencing information (Rosen et al., 2023). Further integration of omics modalities can maximize the number of tokens in the models. Although there are many models for single cell analysis, there are relatively few models for spatial sequencing (Schaar et al., 2024; Choi et al., 2024; Wang et al., 2025). None of the current models can integrate the aforementioned datasets with imaging and metabolomic data.

Currently, zero-shot foundational models have not been shown to reliably outperform advanced machine learning methods such as scVI, or classic logistic regression (Kedzierska et al., 2023; Boiarsky et al., 2023). With models already offering fine-tuning (Cui et al., 2024), it is a question of time until robust task-specific foundation models become available. The short latency between the wide adoption of large-language models by the end of 2022 and the proposal of single-cell foundation models just months later is remarkable. Therefore, advances in large-language models are continuously implemented in single-cell foundation models. One such advance is the recent introduction of byte latent transformers that show improved scalability and robustness with respect to previous tokenization-based models (Pagnoni et al., 2024).

Biological phenomena occur in living systems, making the interpretation of machine learning results dependent on domain-specific knowledge and an understanding of the physiological context during data acquisition, including factors such as species, sex, and age. Developing truly universal foundation models will require addressing these and other unseen variables, all while demanding extensive training datasets and significant computational resources. Just as large-language models face limitations with underrepresented languages and cultural contexts, single-cell models will require time to bridge existing gaps. Until then, machine learning will continue to transform biology—not always by directly solving complex biological problems, but often by addressing related challenges. As the mathematician George Pólya demonstrated (Polya, 2014), tackling adjacent problems can make seemingly intractable questions solvable, paving the way for progress in biology.
